# Comparison of user groups' perspectives of barriers and facilitators to implementing electronic health records: a systematic review

**DOI:** 10.1186/1741-7015-9-46

**Published:** 2011-04-28

**Authors:** Carrie Anna McGinn, Sonya Grenier, Julie Duplantie, Nicola Shaw, Claude Sicotte, Luc Mathieu, Yvan Leduc, France Légaré, Marie-Pierre Gagnon

**Affiliations:** 1 Research Centre of the Centre Hospitalier Universitaire du Québec, Québec, QC, Canada; 2 Department of Social and Preventive Medicine, Université Laval, Québec, QC, Canada; 3 Health Informatics Institute, University of Algoma, Sault-Sainte-Marie, ON, Canada; 4 Department of Health Management, Université de Montréal, Montréal, QC, Canada; 5 Department of Nursing, Université de Sherbrooke, Québec, QC, Canada; 6 Department of Family Medicine, Université Laval, Québec, QC, Canada; 7 Faculty of Nursing, Université Laval, Québec, QC, Canada

## Abstract

**Background:**

Electronic health record (EHR) implementation is currently underway in Canada, as in many other countries. These ambitious projects involve many stakeholders with unique perceptions of the implementation process. EHR users have an important role to play as they must integrate the EHR system into their work environments and use it in their everyday activities. Users hold valuable, first-hand knowledge of what can limit or contribute to the success of EHR implementation projects. A comprehensive synthesis of EHR users' perceptions is key to successful future implementation. This systematic literature review was aimed to synthesize current knowledge of the barriers and facilitators influencing shared EHR implementation among its various users.

**Methods:**

Covering a period from 1999 to 2009, a literature search was conducted on nine electronic databases. Studies were included if they reported on users' perceived barriers and facilitators to shared EHR implementation, in healthcare settings comparable to Canada. Studies in all languages with an empirical study design were included. Quality and relevance of the studies were assessed. Four EHR user groups were targeted: physicians, other health care professionals, managers, and patients/public. Content analysis was performed independently by two authors using a validated extraction grid with pre-established categorization of barriers and facilitators for each group of EHR users.

**Results:**

Of a total of 5,695 potentially relevant publications identified, 117 full text publications were obtained after screening titles and abstracts. After review of the full articles, 60 publications, corresponding to 52 studies, met the inclusion criteria. The most frequent adoption factors common to all user groups were design and technical concerns, ease of use, interoperability, privacy and security, costs, productivity, familiarity and ability with EHR, motivation to use EHR, patient and health professional interaction, and lack of time and workload. Each user group also identified factors specific to their professional and individual priorities.

**Conclusions:**

This systematic review presents innovative research on the barriers and facilitators to EHR implementation. While important similarities between user groups are highlighted, differences between them demonstrate that each user group also has a unique perspective of the implementation process that should be taken into account.

## Background

An interoperable electronic health record (EHR) is defined as a secure and private electronic lifetime record of an individual's key health history and care within the health system [1]. This record is available electronically to authorized health providers and the individual anywhere, anytime in support of high quality care. This record is designed to facilitate the sharing of data across the continuum of care, across healthcare delivery organizations, across time and across geographical areas [1]. The EHR typically contains information such as existing health conditions, physician visits, hospitalizations, test results, and prescribed drugs.

The EHR has the potential to address many of the current challenges healthcare systems face and benefits of its implementation are expected for patients, healthcare professionals, organizations and the general public. EHRs can enable a better quality of care as patients have their essential health data accessible to their different health providers [2,3]. EHRs can provide relevant, timely, and up-to-date information that contributes to knowledge exchange for collaborative decision making among multidisciplinary teams of health care professionals [4-6]. EHRs can also support citizen empowerment and participation in decision-making regarding their health [7], and contribute to creating a safer and more efficient healthcare system [7-11]. Canadian policy makers recognize the importance of the EHR [1] and are currently working in partnership with federal, provincial, and territorial governments and an interprovincial agency aimed at coordinating EHR implementation efforts across Canada - Canada Health Infoway- to develop an ambitious project for its implementation [12]. However, EHR implementation in Canada currently lags behind other industrialized countries [13-15]. A recent study found that only 37% of Canadian family physicians use EHRs, ranking Canada last among the 11 countries surveyed [16].

Decision-makers need scientific evidence on the favorable conditions allowing optimal implementation of EHR in specific contexts; however, these data are currently lacking [17]. The EHR implementation process is influenced by many factors: at the micro-level by interpersonal factors such as individuals' attitudes and concerns and the material properties of EHR technology; at the meso-level by the operational aspects of implementation such as readiness and resources; and at the macro-level by socio-political forces. However, few systematic reviews have been conducted to investigate the barriers and facilitators to EHR implementation and the majority of these studies have focused on health care professionals, particularly physicians [18]. While the comparisons of the perspectives of various professional groups have been reported in scientific literature, these results have not yet been synthesized [19]. As greater interdisciplinary practice is encouraged in the health care system [20], understanding and comparing the perspectives of each user group is essential to the successful implementation of EHRs.

This study is a systematic review of the perceived barriers and facilitators of interoperable EHR implementation whose ultimate goal is to answer real challenges decision-makers face. More specifically, the objectives were to categorize, synthesize, and compare the perspectives of targeted groups of users (public, patients, health care professionals and managers) and to underline factors influencing EHR implementation specific to each user group.

## Methods

### Search strategy

Using a literature search strategy developed by an information specialist (available upon request), the following databases were searched to identify relevant papers published between 1999 and 2009: PubMed, EMBASE, CINAHL, Business Source Premier, Science Citation Index, Social Sciences Citation Index, Cochrane Library, ABI/Inform, and PsychINFO. The research team identified applicable articles and verified their inclusion in the search results in order to ensure the sensitivity of the search strategy. References from included studies were also assessed.

### Selection criteria

The studies included in this review met the following criteria:

#### Empirical

The studies had an empirical study design, either qualitative, quantitative, or mixed-methods. The data collection process was clearly stated and research strategies and measurement tools were present. As such, editorials, comments, position papers, and unstructured observations were excluded.

#### Interoperable EHR

The targeted intervention was the implementation of a general, interoperable EHR. Studies that focused on only a sub-function of an EHR (such as clinical reminders) were excluded, as were systems related to a specific disease and those that did not include communication with a patient's health record (such as physician-laboratory information systems).

#### User perspective

Users' perspective of EHR implementation was documented. User groups included in this study were health professionals (physicians, nurses, pharmacists, and medical archivists), managers and patients.

#### Barriers and facilitators

Barriers and facilitators to EHR implementation were clearly mentioned in the study results. We did not restrict the search to studies reporting these as their main objective; however, all included studies provided data based upon empirical evidence for either of these two factors.

#### Implementation

The study was based upon an actual EHR implementation experience. As such, studies not focusing on a "real life" EHR project, such as opinion surveys, were excluded.

#### Country

Only studies that took place in Canada or in countries with comparable socio-economic levels to Canada were included [21]: Australia, Austria, Belgium, Denmark, Finland, France, Germany, Greece, Iceland, Ireland, Italy, Japan, Luxembourg, the Netherlands, New Zealand, Norway, Spain, Sweden, Switzerland, United Kingdom, and the United States.

When a study was described by more than one publication and presented the same data, only the most recent publication was included. However, if new data were presented in multiple publications pertaining to the same study, all were included. Studies in all languages were considered.

### Screening and data extraction

One reviewer (SG) initially screened all titles and abstracts of references captured by the search strategy and two independent reviewers (SG and CAM or JD, mediated by MPG) reviewed the titles and abstracts retained by SG. Full texts of the final selection of studies were reviewed by SG, validated by CAM, and mediated by MPG.

Data extraction was undertaken using a validated data extraction grid, developed through previous research related to the classification of barriers and facilitators to the implementation of shared decision-making in healthcare settings [22-25]. The data extraction grid was created using both inductive and deductive methods, following established theoretical concepts [18,26-30], particularly the Technology Acceptance Model [27] and the Diffusion of Innovations Theory [28]. Recently, the research team adapted and validated this data extraction grid to classify the reported barriers and facilitators to Information and Communication Technologies adoption in healthcare settings [31]. We adapted this most recent version of the grid, adding other emergent categories relevant to EHR implementation during the data extraction process; however, we did not remove any existing categories. To consult the final data extraction grid, see additional file 1.

The data extraction grid was reproduced in the NVivo qualitative data analysis software **N'Vivo (version 7) (Qualitative Research Solution, Pty Ltd., Australia **[32]. All publications were uploaded into NVivo and two reviewers independently read publications and coded sections of text that represented a relevant barrier or facilitator to the implementation of interoperable EHR. Data were also abstracted concerning: year of publication, country of origin, EHR technology implemented, type of participants, study design (quantitative, qualitative, or mixed-methods), theoretical framework (present or absent), data collection methods, implementation level (national, regional, or local), and organization type (single or multiple).

### Study quality assessment

Study quality was appraised using a mixed methods research scoring system developed by Pluye *et al. *[33], which proposes evaluation criteria for quantitative, qualitative, and mixed-methods studies. All included studies were screened for quality and relevance by two researchers and no studies were excluded based upon their scores (results for quality assessment available upon request).

## Results

### Included studies

**Figure 1:**
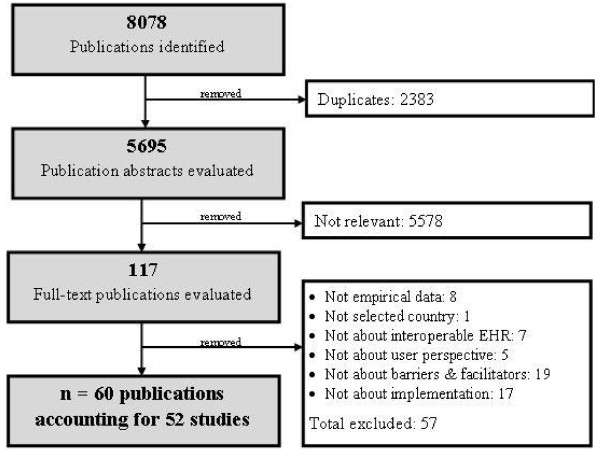
**Study selection flow diagram**.

Eight thousand, seventy-eight references were initially retrieved from bibliographic databases. After controlling for duplicates, the 5,695 remaining titles and abstracts were screened, of which 117 publications were retained for full-text review. After applying the inclusion criteria, 57 of these publications were excluded. The review, therefore, included 60 publications [34-93] corresponding to 52 studies. The number of studies included at various stages of the review process is described in a study selection flow diagram (Figure 1).

### Characteristics of included studies

The characteristics of included studies are summarised in Additional file 2. The most frequent types of technology covered were: electronic health records (EHR) (n = 23 studies) [39,40,42,45,47,49,54-59,62,65-68,70,72,75-77,79,81,90,92]; electronic medical records (EMR) (n = 19) [35,43,44,46,48,51,52,60,61,64,69,71,73,74,78,82,83,86,88]; electronic patient records (EPR) (n = 5) [50,53,87,91,93] and computer-based/computerized patient records (n = 2) [36,63]. Other forms of technology were each represented by one study: computerized patient information system [80], computerized medical records [84], electronic records management [34], personal health records [38], portable computers [89], smart card [85], and summary care records [37,41].

The majority of the studies took place in North America (n = 31, 59.6%), of those 6 are from Canada (11.5%) [35,39,50,66,85,89] and 28 (48.1%) from the United States [34,38,40,42,45,47,48,54-57,59,61-65,69-71,73,76-78,82,83,86,92]. A large number of studies (n = 17, 32.7%) were conducted in European countries: United Kingdom (n = 10) [37,41,53,58,67,68,72,79,81,90], Norway (n = 5) [46,50,60,87,88], Sweden (n = 3) [36,51,52], Denmark (n = 3) [75,91,93], Greece (n = 1) [49] and Spain (n = 1) [74]. Two Australian (3.8%) [80,84] and two Japanese studies [43,44] were also included in this systematic review. More than half of the studies were published since 2006 (n = 29, 55.8%).

The study participants were wide-ranging. Seventeen studies (32.7%) nearly exclusively involved physicians [35,40,45,46,55,57,59,62,65,67,69,71,74,76,82,83,86,87,91-93] while another 17 studies concerned a variety of health care professionals, most notably a combination of physicians, nurses, and administrative staff, and less commonly pharmacists, midwives, and social workers [36,37,44,48-52,54,56,60,64,70,73,80,85,88,90]. Ten studies (19.2%) primarily involved participants in management positions, including health information managers, hospital directors, various clinicians, and representatives from EHR vendors and Information Technology (IT) consulting firms [34,39,42,43,47,53,58,61,63,78]. Patients and the public were the focus of eight studies (15.4%) [38,41,66,68,72,75,77,79,81,84,89]. A study by Greenhalgh *et al. *included a publication pertaining to health professionals [37] and another pertaining to patients [41].

Nearly half of the studies (n = 25, 48.1%) were quantitative, primarily using surveys. Twenty-two studies (42.3%) had a qualitative research approach, using one or more of the following methods for data collection: interviews, focus groups, open-ended questionnaires, observation, and document analyses. Six studies (11.5%) used a mixed approach, such as a combination of surveys with open and closed questions, interviews, and focus groups. Less than a quarter of the studies (n = 11, 21.2%) included a theoretical framework.

The level of implementation varied. Over half the studies (n = 29, 55.8%) were locally implemented; others were regional (n = 15, 28.8%) or national (n = 8, 15.4%) implementation projects. Thirty-four studies involved multiple organizations (65.4%), while 18 took place within a single organization.

### Factors common to all user groups

The final categorization of barriers and facilitators to EHR implementation is presented in Additional file 3, Table S1. Nearly all factors were perceived as being a barrier by some and a facilitator by others. It is worthwhile to mention that more barriers than facilitating factors were mentioned overall. Ten factors were common among all EHR user groups, as discussed below and summarized in Table 1. More details about the barriers and the facilitators are provided in additional file 4.

**Table 1:** Electronic health record implementation factors common to all user groups

Factor	User groups (number of studies)				Number of studies (%)
		
	Physicians	Health care professionals	Managers	Patients	
Design or technical concerns	9	9	3	1	22 (42.3)
Privacy and security concerns	4	5	4	8	21 (40.4)
Cost issues	8	3	7	1	19 (36.5)
Lack of time and workload	7	6	3	1	17 (32.7)
Motivation to use EHR	3	7	2	4	16 (30.8)
Productivity	4	5	3	2	14 (26.9)
Perceived ease of use	3	6	2	2	13 (25.0)
Patient and health professional interaction	3	4	1	4	12 (23.1)
Interoperability	2	2	3	3	10 (19.2)
Familiarity, ability with EHR	2	2	2	3	9 (17.3)

#### Design or technical concerns

Issues related to the technical aspects of EHR were the most frequently mentioned factor, cited by 22 of the 52 included studies (42.3%). This factor was nearly always considered a barrier to EHR implementation. The most frequently mentioned barriers were the technical limitations related to software or hardware, and system problems (that is, slow system speed, unplanned downtime, and so on) [36,46,54-57,65,70,73,75,87,88]. Concerns that the system would become obsolete were also mentioned [40,48].

#### Perceived ease of use

Overall, ease of use was perceived as being both a barrier and facilitator to EHR implementation and was closely associated with design and technical issues. Where systems were reported as user-friendly, participants tended to perceive EHRs as easy to use and a valuable tool to facilitate work processes [51,73,77,79,85]. However, when systems were not adapted to the needs or abilities of the users, studies reported participants as perceiving the EHR system as being difficult to use [34,36,37,40,78,80]. Other issues were related to the lack of understanding of EHR features [65] or confusing screens, options, and navigational aids [78].

#### Interoperability

Interoperability, that is the exchange in health data involving more than one organization and/or setting of care [4], was cited more often as a barrier than as a facilitator to EHR implementation. Generally, inadequate interfacing with other IT systems was perceived as a barrier by users [34,37,47,74,75,92], and in some cases led to negative outcomes. For example, Ferris *et al. *[34] found that when external connectivity to laboratories for test results was not fully implemented in medical practices, both EHR and paper-based systems were required to manage test results, which led to erratic use of the EHR by physicians.

#### Privacy and security concerns

Privacy and security was the second-most mentioned factor in the systematic review, cited by 21 of the 52 included studies (40.4%). Studies pertaining to physicians and health professionals perceived this factor as a barrier to EHR implementation, while studies related to managers and patients presented this factor as both a barrier and a facilitator. Studies concerning all user groups expressed general concerns that EHR use may compromise the security or confidentiality of patient information [38,41,42,47-49,53,57,58,65,71-73,79,82-84,86,90], either within the health center or through electronic links to other organizations. One study with physicians highlighted fears of loss of personal and professional privacy [83] and a patient study specifically mentioned concerns about potential commercial use of health data [79]. Overall, patients appear to have a more nuanced point of view on privacy issues. While four studies raised concerns [38,41,79,81,84], five studies also reported that confidentiality and security were issues of little concern to their patient participants [66,68,75,77,79].

#### Cost issues

Cost issues were overwhelmingly considered a barrier to EHR implementation (19 studies, 36.5%). Studies pertaining to health care professionals and patients highlighted more general concerns about high costs [44,48,51,52,72], whereas studies related to managers and physicians were more inclined to mention specific issues such as lack of resources and funding [39,40,42,45,62,78], high start-up costs [40,47,57,59,69,78], high on-going maintenance costs [57,59,69], and uncertainty about return on investment [40,47].

#### Productivity

Loss of clinical productivity and decreased job performance, particularly during the transition period to an EHR system, were perceived as barriers [48,55-57,59,61,62], and concerns about consequent costs were often associated with this factor [57,59,78]. However, this issue was more often perceived as a facilitator in studies related to health professionals, managers, and patients, which reported EHRs as positively influencing workplace efficiency and communication [43,47,56,64,66,85,88,89]. Kossman [56] highlighted how productivity may be perceived as both a barrier and a facilitator to EHR implementation. Nurses in this study stated that increased time spent interacting with the EHR system decreased their job performance because they spent less time with patients; however, this same study also found that nurses perceived EHRs as improving workplace productivity due to better access to and organization of patient care information.

#### Familiarity and ability with EHR

Studies that presented patients' point of view found that they were generally familiar with computers [72,77] and perceived EHRs as easy to access and use [68,77]. However, studies related to physicians, health professionals, and managers perceived this factor as a barrier. For instance, managers expressed concerns about patient computer literacy [39] or general lack of knowledge about EHRs [42], whereas health professionals perceived themselves as lacking computer experience [37,74,93].

#### Motivation to use EHR

This factor was cited as both a barrier and a facilitator. Resistance to change was the primary source of de-motivation in studies among health care professionals [36,90], while a lack of knowledge or interest in EHRs was reported in a study on patients [68]. Facilitators for all user groups were generally reported as positive attitudes toward the continued use and benefits of EHRs [46,60,68,73,75,80,85,89,91].

#### Patient and health professional interaction

The studies involving health care providers and patients reported that EHRs tended to be perceived as negatively impacting the relationship between patients and health care providers. The most cited interaction change reported by clinicians and managers was a loss of both physical and relational contact with the patient due to interaction with the technology [35,54,56,82,83,86] and perceptions that EHRs interrupt rather than support nurses' ability to provide direct patient care [60]. Patients concerns, however, focused on changes to the patient-physician relationship, such as receiving bad news about their health electronically rather than in person [79], or EHRs being used by physicians to selectively choose their patients [41]. Two patient studies reported that EHRs did not affect the patient-clinician relationship [84,89].

#### Lack of time and workload

Studies related to physicians, other health care professionals and managers cited lack of time and workload as important barriers to EHR implementation. Studies involving health care professionals made more general statements about heavy workloads [41,49] and EHR use as being time-consuming [36,48,49,73,80]. Studies concerning physicians tended to give more detailed reasons, such as the lack of time to acquire, implement and learn to use EHRs [57,59,69,93] and concerns that EHR implementation would take time away from physicians' clinical tasks [83,93]. Studies about managers expressed concern about EHR use increasing physician workload [43,78]. Only one patient study cited this factor, stating that EHRs may be a valuable tool to reduce clinicians' workload [89].

### Factors specific to each user group

Studies related to physicians mentioned two barriers specific to this group: participation of end-users in the selection and planning, and physician salary status, that is, the fee for service remuneration of physicians. The most-cited factors influencing EHR implementation (by at least 8 of 17 studies related to physicians) were also barriers: design and technical issues and cost issues.

Overall, studies concerning health professionals mentioned a greater set of factors unique to their user group: trialability, observability, evidence regarding the benefits of EHR, scientific quality of the EHR resources, ethical issues, attitudes of colleagues about EHR, support and promotion of EHR by colleagues, and competition. The most-cited factors for this user group (8 of 17) were design or technical issues and perceived usefulness.

There were fewer studies related to managers' or patients' perspectives. The most-cited factor in studies about managers (5 of 10) was cost, and the top-cited factors for patients (4 of 8) were perceived usefulness, privacy and security concerns, accuracy, risk-benefit equation, motivation to use EHR, and patient and health professional interaction. Factors unique to patients were autonomy and patients' attitudes and preferences towards EHR. Studies related to patients tended to regard familiarity and ability with EHR as facilitating factors. In fact, studies focusing on patients' perspective reported facilitating factors in a larger proportion (61%) than studies related to health care professionals (30%), physicians (23%) and managers (21%).

## Discussion

The main findings of our systematic review suggest that 10 implementation factors are relevant to all user groups, and that among these factors design and technical concerns, cost issues, privacy and security concerns, lack of time and workload are among the most-cited. Systematic reviews by members of our team [31], Boonstra and Broekhuis [94] and Castillo *et al. *[95] support these findings. Their results confirm that financial, time-related, and technical barriers are the most-cited barriers to EHR acceptance and adoption. As Boonstra and Broekhuis also point out, these "primary" barriers are related to pressing first-hand problems related to EHR use, and that secondary factors related to social, psychological, and change processes may be less-mentioned in the literature. Our study highlights many individual, human, and organizational environment factors, such as motivation to use EHR and issues related to patient and health professional interaction, as well as many primary barriers, such as ease of use and productivity, that may need to be addressed simultaneously to encourage optimal EHR implementation.

This systematic review aimed to uncover not only similarities but also differences among user groups. Overall, studies involving physicians and health professionals provided data on the widest variety of factors. As opposed to the other user groups, studies related to patients cited few factors in the Organization category owing to the fact that patients are generally not privy to organizational processes. While accuracy of information contained within the EHR was one of the most-cited factors for patients [66,68,72,79,84,89], accuracy was only mentioned in one other study, pertaining to managers [63]. Moreover, patients were the only user group to identify facilitating factors in a larger proportion than barriers and to consider autonomy, that is health empowerment and improved health self-management, as a positive EHR implementation factor [38,72,79].

Results from included studies on physicians, health professionals, and managers indicate the importance of eight organizational factors: practice size, change in tasks, human resources regarding IT support, training, management, relationship between administration and health professionals, choice of the EHR system, and interorganizational relations as influencing EHR implementation. These factors highlight the particular challenges these user groups face in their work environments. In studies where adequate technological support and training was provided, these factors tended to be perceived as facilitators, while studies which reported inadequate or no IT support or training tended to conclude that these factors were barriers to EHR implementation. Similarly, the managerial approach can be key to EHR implementation: forcefully implementing EHR contributed to failure while adopting a bottom-up approach fostered enthusiasm, dedication, and commitment from individuals, thus contributing to successful implementation [63]. Improving change management processes is a promising solution to overcoming these barriers since adequate change management can mediate other identified barriers [94].

User groups could also perceive the same factor differently. For instance, studies related to physicians, health professionals, and managers differently interpreted management involvement. Physician studies reported that a barrier to EHR implementation was the perception that the EHR system acted as a control mechanism allowing management to infringe on physicians' professional autonomy [91,93]. Studies on health professionals, however, tended to consider poor organization management practices as barriers to EHR implementation, such as a top-down leadership approach [50,64], poor timing [36], and providing inadequate resources to support implementation [37,90]. This same user group positively perceived reflexive management approaches [37,50], prioritization and driving by the management team [51], and voluntariness [85] as facilitators. One study involving managers reported poor management techniques as a barrier that exacerbated implementation challenges and fostered passive resistance to EHR implementation [61].

Our study also raises a little-studied issue in that physicians may perceive their professional autonomy to be threatened or harbored by EHR implementation. Professional autonomy may generally be defined as 'professionals' having control over the conditions, processes, procedures, or content of their work according to their own collective and, ultimately, individual judgment in the application of their profession's body of knowledge and expertise professional privacy' [96]. Our systematic review found studies expressing concern about EHR systems infringing on physicians' personal and professional privacy [83] and acting as management control mechanisms [91,93]. This finding echoes innovative research by Walter and Lopez [96] pointing out that physicians' perceived threat to professional autonomy has a significant negative impact on both perceived usefulness and intention to use an information technology. However, another study reported that general practitioners believe that: 'contemporary health care requires a radical change in how confidentiality and privacy are defined (from a property of the individual doctor-patient relationship, mediated by the human qualities of the doctor, to a property of the system as a whole, mediated by technical and operational security measures)' [37]. This issue should be explored in further research, particularly in the Canadian context.

A lack of uniform EHR standards, at local, regional, or national levels, was a clearly stated barrier in studies pertaining to physicians and managers [47,57,59,63,69]. Lack of standardization may contribute to physicians' and managers' disorientation when choosing an EHR system. Studies show that they were often inexperienced [35] and had difficulty selecting among many potential systems [92], which in some cases led to an inability to find an appropriate system or the implementation of an ill-suited system [47,61,62]. Certain studies also highlighted users' lack of confidence in EHR vendors, such as fears that vendors may provide inadequate support [35] or go out of business [92]. Gans *et al. *[62] suggested that actions are needed to make the EHR decision process easier for practices, such as certification for EHR vendors and educational programs on how to select and implement an EHR system.

This systematic review adds to the current evidence that individual, human, and organizational barriers remain challenges that must be addressed in an innovative manner, according to the particular needs of each implementation project and each user group. Active participation of end-users in EHR implementation is a promising strategy since it allows decision-makers to consider users' perspectives, gain their support, and adapt the technology to users' needs [97].

Understanding the facilitators to EHR implementation is also key to successful implementation. Our study highlights two factors, perceived usefulness and motivation to use EHRs, as chief facilitators to EHR implementation. These two factors are closely related and should be considered when implementing EHRs since a positive perception of its usefulness increases users' motivation to use it [63].

## Study limitations and future research

One potential limitation of this systematic review pertains to the categorization of included studies according to four EHR user groups, based on the main group represented in each study, for the purpose of establishing comparisons between groups. This method is somewhat limited because most studies, especially those related to health care professionals, involved multiple user groups and generally gave overall group results rather than information specific to each individual group. As such, it is possible that the results presented are not completely mutually exclusive across each group of EHR users. Furthermore, we did not contact the authors of the studies to confirm that we had categorized their findings in appropriate ways, which may constitute a limitation as mentioned by Boonstra and Broekhuis [94]. However, we do not think that contacting the authors would have changed the results of this study or the developed taxonomy.

## Conclusions

This systematic review presents an integrative and comprehensive summary of four main EHR user groups' perceptions of the barriers and facilitators related to EHR implementation. This is the first systematic review on barriers and facilitators to EHR implementation that includes the patients' perspective and compares it with those of physicians, health professionals and managers. Our study is the first to summarize and compare the perceptions of different user groups and to present an overall perspective of the barriers and facilitators that are common or unique to each group.

EHR implementation is a complex and multi-dimensional process that is influenced by many technical, individual, human, and organizational factors. After examining the similarities between user groups, we found that physicians, health care professionals, and managers share many common factors. While similarities are drawn between groups, inter-group differences also show how the unique perspective of each user group needs to be taken into account. We propose that future decision-making regarding EHR implementation should draw upon these innovative findings and consider EHR users' wide-ranging perspectives of the barriers and facilitators to EHR implementation. Our next research steps include the validation of these findings specifically for the Canadian context, through a Delphi study among Canadian EHR users, thus complementing this systematic review with knowledge about the specific Canadian context.

## Abbreviations

EHR: electronic health record; IT: information technology.

## Competing interests

The authors declare that they have no competing interests.

## Authors' contributions

MPG, FL, JD, NS, CS, YL and LM participated in the study design and received research funding. MPG, SG, CAM, JD and FL contributed to study screening and selection. MPG, SG, CAM and JD participated in the data extraction process. CAM and SG drafted the manuscript. All authors critically reviewed and approved the final manuscript.

## Pre-publication history

The pre-publication history for this paper can be accessed here:

http://www.biomedcentral.com/1741-7015/9/46/prepub

## Supplementary Material

Additional file 1 **Data extraction grid: Facilitating factors or barriers related to EHR implementation**. Click here for file

Additional file 2 **Characteristics of included studies, per EHR user group**. Click here for file

Additional file 3 **Table S1. Factors perceived as barriers (B) or facilitators (F) influencing electronic health record implementation, per user group**. Click here for file

Additional file 4 **Barriers and facilitators to EHR implementation, per study (numbers in bold refer to the extraction codes in Additional file **1). Click here for file
