# Supplementary material for: Comparison of user groups' perspectives of barriers and facilitators to implementing electronic health records: a systematic review
Source: BMC Med. 2011 Apr 28;9:46. doi: 10.1186/1741-7015-9-46 (PMC3103434; doi:10.1186/1741-7015-9-46)
Supplement: Additional file 1 — Data extraction grid: Facilitating factors or barriers related to EHR implementation. [file 1741-7015-9-46-S1.PDF]

Additional file 1. Data extraction grid: Facilitating factors or barriers related to EHR implementation

**1 Factors related to EHR characteristics**
1.1 Design and technical concerns
1.2 Characteristics of innovation
1.2.1 Perceived usefulness
1.2.2 Compatibility (with work process)
1.2.3 Perceived ease of use
1.2.4 Trialability
1.2.5 Observability
1.3 System reliability or dependability
1.4 Interoperability
1.5 Legal issues
1.5.1 Privacy and security concerns
1.5.2 Medicolegal issues
1.6 Evidence regarding benefits of IT
1.7 Validity of the resources
1.7.1 Scientific quality of the EHR resources
1.7.2 Satisfaction about content available (completeness)
1.7.3 Content appropriate for the users (relevance)
1.7.4 Accuracy (improved OR errors, omissions)
1.7.5 Quality standard
1.8 Participation of end-users in the design
1.9 Cost issues
1.10 Environmental issues
1.11 Ethical issues
1.12 Productivity
**2 Individual factors: knowledge, attitude and socio-demographic characteristics**
2.1 Knowledge
2.1.1 Awareness of the objectives and/or existence of the EHR
2.1.2 Familiarity, ability with EHR
2.1.3 Familiarity with technologies in general
2.2 Attitude
2.2.1 Patient agreement with the EHR
2.2.1.1 Risk-benefit equation (perception)
2.2.1.2 Confidence in EHR developer or vendor
2.2.1.3 Autonomy (health empowerment)
2.2.1.4 Impact on clinical uncertainty
2.2.1.5 Time issues
2.2.1.6 Outcome expectancy (use of the EHR leads or not to desired outcome)
2.2.1.7 Motivation/inertia to use EHR (readiness)/resistance to use the EHR
2.2.1.8 Self-efficacy (belief in one's competence to use the EHR)

2.2.1.9 Impact on professional security
2.2.2 Health professionals' agreement with EHR
2.2.2.1 Risk-benefit equation (perception)
2.2.2.2 Confidence in EHR developer or vendor
2.2.2.3 Autonomy
2.2.2.4 Impact on clinical uncertainty
2.2.2.5 Time issues
2.2.2.6 Outcome expectancy (use of the EHR leads or not to desired outcome)
2.2.2.7 Motivation/inertia to use EHR (readiness)/resistance to use the EHR
2.2.2.8 Self-efficacy (belief in one's competence to use the EHR)
2.2.2.9 Impact on professional security
2.2.3 Managers' agreement with EHR
2.2.3.1 Risk-benefit equation (perception)
2.2.3.2 Confidence in EHR developer or vendor
2.2.3.3 Autonomy
2.2.3.4 Impact on clinical uncertainty
2.2.3.5 Time issues
2.2.3.6 Outcome expectancy (use of the EHR leads or not to desired outcome)
2.2.3.7 Motivation/inertia to use ICT (readiness)/resistance to use the EHR
2.2.3.8 Self-efficacy (believes in one's competence to use the EHR)
2.2.3.9 Impact on professional security
2.2.4 Agreement with EHRs in general (Welcoming/resistant)
2.2.5 Attitude at work (abilities, qualities)
2.3 Socio-demographical characteristics
2.3.1 Age
2.3.2 Gender
2.3.3 Experience
2.3.4 Ethnicity
2.3.5 Other

3 External factors: human environment

3.1 Factors associated with patients
3.1.1 Patients' attitudes and preferences towards EHR
3.1.2 Patient and Health Professional interaction
3.1.3 Applicability to the characteristics of patients
3.1.4 Others factors associated with patients
3.2 Factors associated with peers
3.2.1 Attitude of colleagues about EHR
3.2.2 Support and promotion of EHR by colleagues
3.2.3 Others factors associated with peers

4 External factors : organisational environment

4.1 Internal environment
4.1.1 Characteristics of the structure of work
4.1.1.1 Setting of care (hospital, outpatient, GP)
4.1.1.2 Practice size

- 4.1.1.3 Status (University/other, private/public)
- 4.1.1.4 Physician salary status and reimbursement
- 4.1.1.5 Workforce issues (shortage, retention)

4.1.2 Nature of work
- 4.1.2.1 Lack of time and workload
- 4.1.2.2 Work flexibility
- 4.1.2.3 Relations among colleagues
- 4.1.2.3.1 Team spirit, cohesion
- 4.1.2.3.2 Competition and relation between different health professionals (including role boundaries)
- 4.1.2.4 Change in task
- 4.1.2.5 Professional culture

4.1.3 Skills (Staff)
- 4.1.3.1 Presence and influence of "champions"
- 4.1.3.2 Leadership
- 4.1.3.3 Computer skill
- 4.1.3.4 Workforce stability

4.1.4 Resources
- 4.1.4.1 Resources available
- 4.1.4.2 Materials resources (access to EHR)
- 4.1.4.3 Human resources (IT support, other)

4.1.5 Organization factors
- 4.1.5.1 Training
- 4.1.5.2 Innovation culture
- 4.1.5.3 Management (strategic plan to implement EHR)
- 4.1.5.4 Communication (included promotional activities)
- 4.1.5.5 Relation between administration and health professionals
- 4.1.5.6 Participation of end-users in the implementation strategy
- 4.1.5.7 Organizational support
- 4.1.5.8 Incentive structures
- 4.1.5.9 Readiness
- 4.1.5.10 Choice of the EHR system
- 4.1.5.11 Other organisational or cultural aspects
- 4.1.5.12 Supporting "communities of practices"

4.2 External environment
- 4.2.1 Financing of EHR / Financial support
- 4.2.2 Interorganisational relations
- 4.2.3 Health care policies and socio political context
