# Supplementary material for: Comparison of user groups' perspectives of barriers and facilitators to implementing electronic health records: a systematic review
Source: BMC Med. 2011 Apr 28;9:46. doi: 10.1186/1741-7015-9-46 (PMC3103434; doi:10.1186/1741-7015-9-46)
Supplement: Additional file 2 — Characteristics of included studies, per EHR user group. [file 1741-7015-9-46-S2.PDF]

Additional file 2: Characteristics of included studies, per EHR user group

Study	Country	Technology*	Participants	Study design	Theoretical frame-work	Data collection	Level of implementation	Organisation
**Physicians**								
Alonso 2004 [74]	Spain	EMR	Physicians	Quantitative	-	Survey	Local	Single
Audet 2004 [69]	USA	EMR	Physicians	Quantitative	-	Survey	National	Multi
Christensen 2008 [87]	Norway	EPR	Physicians	Mixed	-	Observation, focus groups, & questionnaires	Local	Multi
Davidson 2007 [92]	USA	EHR	Physicians	Qualitative	Yes	Action research methodology	Local	Single
DesRoches 2008 [40]	USA	EHR	Physicians	Quantitative	-	Survey	National	Multi
Gadd 2001 [82, 83, 86]	USA	EMR	Physicians & patients	Quantitative	-	Survey & interviews	Local	Multi
Gans 2005 [62]	USA	EHR	Physicians	Quantitative	-	Survey	Regional	Multi
Hier 2005 & 2005 [65, 76]	USA	EHR	Physicians	Quantitative	-	Survey	Local	Single
Jensen 2007 [91, 93]	Denmark	EPR	Physicians & nurses	Qualitative	Yes	Interviews, focus groups, & observation	Local	Single
Keddie 2005 [67]	UK	EHR	Physicians	Quantitative	-	Survey	Local	Multi
Lium 2008 [46]	Norway	EMR	Physicians	Qualitative	-	Interviews	Local	Multi
Ludwick 2009 [35]	Canada	EMR	Physicians	Qualitative	-	Interviews	Local	Multi
Menachemi 2006 [59]	USA	EHR	Physicians	Quantitative	-	Survey	Regional	Multi
Russell 2004 [71]	USA	EMR	Physicians	Quantitative	-	Survey	Local	Multi
Sequist 2007 [55]	USA	EHR	Physicians	Quantitative	-	Survey	National	Multi
Simon 2007 [57]	USA	EHR	Physicians	Quantitative	-	Survey	Regional	Multi
Simon 2008 [45]	USA	EHR	Physicians	Quantitative	-	Survey	Regional	Multi

**Health care professionals**								
Auber 2001 [85]	Canada	Smart Card	Health care professionals & patients	Quantitative	Yes	Survey & interviews	Regional	Multi
Boulus 2007 [50]	Canada & Norway	EPR	Physicians, nurses, secretaries, IT staff, & decision-makers	Qualitative		Ethnographic	Local	Single
Chronaki 2007 [49]	Greece	EHR	Health care professionals & patients	Quantitative	-	Questionnaire	Regional	Multi
Crosson 2005 [64]	USA	EMR	Physicians, nurses , medical secretaries, medical assistants, & office managers	Qualitative	-	Interviews & observation	Local	Single
Darbyshire 2004 [80]	Australia	CPIS	Nurses & midwives	Qualitative	-	Focus groups	Regional	Multi
Goddard 2001 [90]	UK	EHR	Health care professionals	Qualitative	Yes	Questionnaire & interviews	Local	Single
Greenhalgh 2008[§] [37]	UK	SCR	Project staff, voluntary sector staff, & patients	Mixed	Yes	Interviews, focus groups, observation, field notes, questionnaires, document analyses, monitoring statistics	Regional	Multi
Kossman 2006 [56]	USA	EHR	Nurses	Qualitative	Yes	Survey, interviews, & observation	Local	Multi

Laerum 2004 [88]	Norway	EMR	Physicians, nurses, & medical secretaries	Quantitative	-	Survey	Local	Single
Likourezos 2004 [73]	USA	EMR	Physicians & nurses	Quantitative	-	Survey	Local	Single
Linder 2006 [54]	USA	EHR	Physicians, nurses, & others not specified	Quantitative	-	Survey	Regional	Multi
Lium 2006 [60]	Norway	EMR	Physicians, nurses, & medical secretaries	Quantitative	-	Survey	Local	Single
Moody 2004 [70]	USA	EHR	Nurses	Quantitative	-	Survey	Local	Single
Ochieng 2006 [44]	Japan	EMR	Health care professionals	Quantitative	Yes	Survey	Regional	Multi
Ovretveit 2007 [51, 52]	Sweden	EMR	Health care professionals	Qualitative	Yes	Interviews, observation, & document analyses	Regional	Single
Rahimi 2008 [36]	Sweden	CPR	Physicians, nurses, social workers, administrator, & pharmacists	Qualitative	-	Interviews & document analyses	Local	Single
Randeree 2007[48]	USA	EMR	Physicians, physicians' assistants, nurses, office staff	Qualitative	-	Interviews	Regional	Multi
**Managers**								

Ferris 2009 [34]	USA	ERM	Managers, practice managers, medical directors, & physicians	Qualitative	Yes	Survey & interviews	Local	Multi
Houser 2008 [42]	USA	EHR	Health information management directors & hospital administrative personnel	Quantitative	-	Survey	Regional	Multi
Lorence 2005 [63]	USA	CPR	Health information managers	Quantitative	-	Survey	National	Multi
Mannan 2006 [58]	UK	EHR	Physicians, nurses, managers, & receptionists	Qualitative	-	Interviews	Local	Multi
Martin 2007 [53]	UK	EPR	Project team (including managers, analysts, & users)	Qualitative	-	Ethnographic	Local	Multi
Miller 2004 [78]	USA	EMR	Physicians, managers, & representatives of EMR vendors, professional medical associations, & IT consulting firms	Qualitative	-	Interviews	Regional	Multi

Scott 2005 [61]	USA	EMR	Clinicians, managers, & project team members	Qualitative	-	Interviews	Local	Multi
Thakkar 2006 [47]	USA	EHR	Health information managers	Quantitative	-	Survey	National	Multi
Urowitz 2008 [39]	Canada	EHR	Chief Executive Officers, chiefs of medicine, nursing, & informatics, managers of health records or health information services, & privacy officers	Quantitative	-	Questionnaire	National	Multi
Yasunaga 2008 [43]	Japan	EMR	Medical institutions	Quantitative	-	Survey	National	Multi
**Patients**								
Bomba 2001 [84]	Australia	CMR	Patients	Qualitative	-	Survey	Local	Single
Dagnone 2006 [89]	Canada	Portable computers	Patients	Qualitative	-	Interviews	Local	Single
Greenhalgh 2008[§] [41]	UK	SCR	Patients & public	Qualitative	Yes	Interviews & focus groups	Regional	Multi
Hassol 2004 [77]	USA	EHR	Patients & physicians	Mixed	-	Survey, interviews, & focus groups	Local	Single
Honeyman 2005 [68]	UK	EHR	Patients	Qualitative	-	Interviews	Local	Single

Keselman 2007 [38]	USA	PHR	Patients	Mixed	-	Survey	National	Multi
Morin 2005 [66]	Canada	EHR	Patients	Mixed	Yes	Interviews & questionnaire	Regional	Multi
Pyper 2004 [72, 79, 81]	UK	EHR	Patients	Mixed	-	Survey, interviews, & focus groups	Local	Single
Zurita 2004 [75]	Denmark	EHR	Patients	Qualitative	-	Interviews, focus groups, & observation	Local	Multi

* CPIS = Computerized patient information system; CMR = Computerized medical record; CPR = Computer-based/computerized patient record; EHR = Electronic health record; EMR = Electronic medical record; EPR = Electronic patient record; ERM = Electronic records management; PHR = Personal health record; SCR = Summary care record

§ The same study by Greenhalgh *et al.* is counted in two different categories, as one publication related to health professionals and the other pertained to patients.
