# Supplementary material for: Comparison of user groups' perspectives of barriers and facilitators to implementing electronic health records: a systematic review
Source: BMC Med. 2011 Apr 28;9:46. doi: 10.1186/1741-7015-9-46 (PMC3103434; doi:10.1186/1741-7015-9-46)
Supplement: Additional file 3 — Table S1. Factors perceived as barriers (B) or facilitators (F) influencing electronic health record implementation, per user group. [file 1741-7015-9-46-S3.DOC]

**Additional file 3 table S1. Factors perceived as barriers (B) or facilitators (F) influencing electronic health record implementation, per user group**

| **Extraction grid reference*** | **Factors** | **User groups ^§^** | | | |
| --- | --- | --- | --- | --- | --- |
|  |  | Physicians | Health care professionals | Managers | Patients |
| **1** | **EHR characteristics** |  |  |  |  |
| **1.1** | Design or technical concerns | Total: 9  9 B [35, 40, 46, 55, 57, 59, 65, 74, 87] | Total: 9  8 B [36, 48, 54, 56, 70, 73, 80, 88]  1 F [51] | Total: 3  2 B [34, 61]  1 F [78] | Total: 1  1 B [75] |
| **1.2** | Characteristics of innovation |  |  |  |  |
| **1.2.1** | Perceived usefulness | Total: 2  1 B [46]  2 F [46, 93] | Total: 8  4 B [44, 60, 64, 73]  6 F [37, 60, 73, 80, 85, 88] |  | Total: 4  1 B [41]  4 F [41, 66, 79, 89] |
| **1.2.2** | Compatibility | Total: 1  1 B [92] | Total: 2  1 B [37]  1 F [85] | Total: 2  2 B [34, 53]  1 F [34] |  |
| **1.2.3** | Perceived ease of use | Total: 3  3 B [40, 46, 65]  1 F [91] | Total: 6  3 B [36, 37, 80]  3 F [51, 73, 85] | Total: 2  2 B [34, 78] | Total: 2  2 F [77, 79] |
| **1.2.4** | Triability |  | Total: 1  1 F [51] |  |  |
| **1.2.5** | Observability |  | Total: 1  1 B [85] |  |  |
| **1.3** | System reliability | Total: 1  1 B [40] | Total: 1  1 B [60] |  | Total: 3  3 B [66, 84, 89]  1 F [89] |
| **1.4** | Interoperability | Total: 2  2 B [74, 92] | Total: 2  1 B [37]  1 F [51] | Total: 3  3 B [34, 47, 78]  1 F [47] | Total: 3  2 B [38, 75]  1 F [66] |
| **1.5** | Legal issues |  |  |  |  |
| **1.5.1** | Privacy and security concerns | Total: 4  4 B [57, 65, 71, 82, 83, 86] | Total: 5  5 B[48, 49, 73, 90] | Total: 4  3 B [42, 53, 58]  1 F [47] | Total: 8  4 B [38, 41, 72, 79, 84]  5 F [66, 68, 75, 77, 79] |
| **1.5.2** | Medicolegal issues | Total: 2  1 B [67]  1 F [40] | Total: 2  2 B [37, 48] |  | Total: 1  1 B [41] |
| **1.6** | Evidence regarding benefits of IT |  | Total: 1  1 B [80] |  |  |
| **1.7** | Validity of the resources |  |  |  |  |
| **1.7.1** | Scientific quality of the EHR resources |  | Total: 1  1 F [85] |  |  |
| **1.7.2** | Satisfaction about content available |  | Total: 3  2 B [88, 90]  1 F [85] |  | Total: 1  1 F [66] |
| **1.7.3** | Content appropriate to the user | Total: 3  3 B [40, 45, 59] | Total : 2  2 B [48, 80] |  | Total: 2  2 B [38, 79]  1 F [72, 79] |
| **1.7.4** | Accuracy |  |  | Total: 1  1 B [63] | Total: 7  2 B [75, 77]  5 F [66, 68, 72, 79, 84, 89] |
| **1.7.5** | Quality standard | Total: 3  3 B [57, 59, 69] |  | Total: 2  2 B [47, 63] |  |
| **1.8** | Participation of end-users in the design | Total : 1  1 B [91] |  |  |  |
| **1.9** | Cost issues | Total: 8  8 B [40, 45, 57, 59, 62, 67, 69, 92] | Total : 3  3 B [44, 48, 51] | Total : 7  7 B [39, 42, 43, 47, 58, 61, 78]  2 F [42, 47] | Total: 1  1 B [72] |
| **1.11** | Ethical issues |  | Total: 2  2 B [37, 54] |  |  |
| **1.12** | Productivity | Total: 4  4 B [55, 57, 59, 62] | Total : 5  2 B : [48, 56]  4 F : [56, 64, 85, 88] | Total: 3  1 B [61]  2 F [43, 47] | Total: 2  2 F [66, 89] |
| **2** | **Individual factors** |  |  |  |  |
| **2.1** | Knowledge |  |  |  |  |
| **2.1.1** | Awareness of the objectives and/or existence of EHR |  |  | Total: 1  1 B [63] | Total: 1  1 B [41] |
| **2.1.2** | Familiarity, ability with EHR | Total: 2  2 B [74, 93]  1 F [74] | Total: 2  2 B [37, 54] | Total: 2  2 B [39, 42] | Total: 3  3 F [68, 72, 77] |
| **2.2** | Attitude |  |  |  |  |
| **2.2.x.1** | Risk-benefit equation |  | Total: 2  2 B [73] |  | Total: 4  2 B [41, 84]  3 F [72, 84, 89] |
| **2.2.x.2** | Confidence in EHR developer | Total: 2  2 B [35, 92] | Total: 2  2 B [37, 48] |  |  |
| **2.2.x.3** | Autonomy |  |  |  | Total: 2  2 F [38, 72, 79] |
| **2.2.x.4** | Impact on clinical uncertainty |  |  | Total: 1  1 F [42] | Total: 1  1 B [41] |
| **2.2.2.5** | Time issues | Total: 1  1 F [87] | Total: 1  1 B [73] |  |  |
| **2.2.x.6** | Outcome expectancy |  | Total: 3  2 B [73, 80]  2 F [70, 73] | Total: 2  1 B [53]  1 F [42] | Total: 1  1 B [41]  1 F [41] |
| **2.2.x.7** | Motivation to use EHR | Total: 3  3 F [46, 87, 91] | Total: 7  2 B [36, 90]  5 F [44, 60, 73, 80, 85] | Total: 2  2 F [42, 47] | Total: 4  1 B [68]  4 F [38, 68, 75, 89] |
| **2.2.4** | Agreement with EHRs in general |  | Total: 1  1 B [51] |  | Total: 2  1 B [41]  2 F [41, 84] |
| **2.3** | Socio-demographical characteristics |  |  |  |  |
| **2.3.1** | Age | Total: 1  [74] | Total: 1  [48] |  |  |
| **3** | **Human environment** |  |  |  |  |
| **3.1** | Factors associated with patients |  |  |  |  |
| **3.1.1** | Patients’ attitudes and preferences towards EHR |  |  |  | Total: 2  2 F [66, 75] |
| **3.1.2** | Patient and health professional interaction | Total: 3  3 B [35, 71, 82, 83, 86]  1 F [86] | Total: 4  4 B [49, 54, 56, 60] | Total: 1  1 F [47] | Total: 4  2 B [41, 79]  2 F [84, 89] |
| **3.2** | Factors associated with peers |  |  |  |  |
| **3.2.1** | Attitude of colleagues about EHR |  | Total: 1  1 B [64] |  |  |
| **3.2.3** | Other factors associated with peer |  | Total: 1  1 B : [37] |  |  |
| **4** | **Organizational environment** |  |  |  |  |
| **4.1** | Internal environment |  |  |  |  |
| **4.1.1** | Characteristics of the structure of work |  |  |  |  |
| **4.1.1.1** | Setting of care (that is, hospital, GP, and so on) |  |  | Total: 2  2 B [42, 43] |  |
| **4.1.1.2** | Practice size | Total: 6  5 B [40, 45, 57, 62, 92]  1 F [69] | Total: 2  1 B [48]  1 F [37] | Total: 2  2 B [47, 78] |  |
| **4.1.1.3** | Status (that is, university/other, public/private, and so on) |  |  | Total: 2  2 B [42, 43] |  |
| **4.1.1.4** | Physician salary status | Total: 2  1 B [35]  1 F [69] |  |  |  |
| **4.1.2** | Nature of work |  |  |  |  |
| **4.1.2.1** | Lack of time and workload | Total: 7  7 B [57, 59, 67, 69, 74, 83, 87, 93]  1 F [91] | Total: 6  6 B [36, 37, 48, 49, 73, 80]  1 F [80] | Total: 3  2 B [43, 78]  1 F [47] | Total: 1  1 F [89] |
| **4.1.2.2** | Work flexibility | Total: 1  1 B [45] |  |  |  |
| **4.1.2.3** | Relationships among colleagues |  |  |  |  |
| **4.1.2.3.1** | Team spirit, cohesion | Total: 2  1 B [93]  1 F [82] | Total: 3  3 B [48, 56, 64] |  |  |
| **4.1.2.3.2** | Competition |  | Total: 2  1 B [80]  1 F [37] |  |  |
| **4.1.2.4** | Change in tasks | Total: 5  3 B [87, 91-93]  1 F [46] | Total: 4  4 B [37, 50, 80, 88]  1 F [50] | Total: 2  2 B [61, 78]  1 F [61] |  |
| **4.1.2.5** | Professional culture |  |  | Total: 1  1 B [61] |  |
| **4.1.3** | Staff skills |  |  |  |  |
| **4.1.3.1** | Presence and influence of “champions” | Total: 1  1 F [93] | Total: 3  1 B [50]  2 F [37, 51] |  |  |
| **4.1.3.2** | Leadership | Total: 2  2 F [46, 92] |  |  |  |
| **4.1.3.3** | Computer skills | Total: 2  2 B [57, 65] | Total : 1  1 B [48] |  |  |
| **4.1.4** | Resources |  |  |  |  |
| **4.1.4.2** | Material resources | Total: 2  2 B [65, 74] | Total: 5  5B [54, 70, 88] |  |  |
| **4.1.4.3** | Human resources (IT support) | Total: 7  6 B [35, 45, 57, 62, 67, 92]  1 F [46] | Total: 5  2 B [37, 48, 49, 51]  3 F [36, 70, 85] | Total: 4  4 B [42, 61, 63, 78] |  |
| **4.1.5** | Organization |  |  |  |  |
| **4.1.5.1** | Training | Total: 3  3 B [55, 67, 74] | Total: 4  4 B [36, 37, 44, 48] | Total: 3  3 B [34, 42, 63] |  |
| **4.1.5.2** | Innovation culture |  | Total: 1  1 B [37] | Total: 1  1 B [47] |  |
| **4.1.5.3** | Management | Total: 2  1 B [91, 93]  2 F [46] | Total: 7  5 B [36, 37, 50, 64, 90]  4 F [37, 50, 51, 85] | Total: 1  1 B [61] |  |
| **4.1.5.4** | Communication |  | Total: 1  1 F [50] |  |  |
| **4.1.5.5** | Relation between administration and health professionals | Total: 1  1 F [46] | Total: 2  1 B [37]  1 F [51] | Total: 1  1 B [61] |  |
| **4.1.5.6** | Participation of end-users in the implementation strategy |  | Total: 3  2 B [36, 51]  2 F [50, 51] | Total: 2  2 B [47, 61] | Total: 1  1 F [89] |
| **4.1.5.7** | Organizational support | Total: 1  1 F [57] |  |  |  |
| **4.1.5.8** | Incentive structures | Total: 1  1 F [57] |  | Total: 1  1 B [78] |  |
| **4.1.5.9** | Readiness |  | Total: 1  1 B [37] |  |  |
| **4.1.5.10** | Choice of the EHR system | Total: 3  3 B [35, 62, 92]  1 F [62] | Total: 1  1 F [48] | Total: 3  3 B [47, 58, 61] |  |
| **4.2** | External environment |  |  |  |  |
| **4.2.1** | Financial support | Total: 2  1 B [92]  1 F [40] | Total: 1  1 F [37] |  |  |
| **4.2.2** | Interorganizational relations | Total: 1  1 F [46] | Total: 1  1 F [37] | Total: 1  1 B [53] |  |
| **4.2.3** | Health care policies and socio political context |  | Total: 1  1 B [37] |  | Total: 1  1 B [84] |

* See additional file 1 for the full data extraction grid.

^§^ The total number of studies may be inferior to the sum of the associated barriers and facilitators, as some studies cited a factor as both a barrier and facilitator
