# Supplementary material for: Comparison of user groups' perspectives of barriers and facilitators to implementing electronic health records: a systematic review
Source: BMC Med. 2011 Apr 28;9:46. doi: 10.1186/1741-7015-9-46 (PMC3103434; doi:10.1186/1741-7015-9-46)
Supplement: Additional file 4 — Barriers and facilitators to EHR implementation, per study (numbers in bold refer to the extraction codes in Additional file 1). [file 1741-7015-9-46-S4.PDF]

Additional file 3. Barriers and facilitators to EHR implementation, per study (numbers in bold refer to the extraction codes in Additional file 1)

Study	Barriers	Facilitators
**Physicians**		
Alonso 2004 [74]	**1.1** Complexity of the program<br>**1.4** Problems with network connection<br>**2.1.2** Lack of typing skills and computer literacy<br>**4.1.2.1** Lack of time<br>**4.1.4.2** Unavailability of computers in the hospital<br>**4.1.5.1** Insufficient training	**2.1.2** Participants who found computers more easy to use were more likely to adopt the EHR<br>**2.3.1** Participants younger than 30 years old were more likely to adopt the EHR
Audet 2004 [69]	**1.7.5:** Lack of local, regional, and national standards<br>**1.9:** Cost of system start-up and maintenance<br>**4.1.2.1:** Lack of time to consider acquiring, implementing, and using a new system	**4.1.1.2** After controlling for salary status, physicians in groups larger than 50 were significantly more likely to be EHR users<br>**4.1.1.4** Mode of compensation (salary)
Christensen 2008 [87]	**1.1** Redundancy of information in patient records<br>**4.1.2.1** A shift in administrative workload from health secretaries to physicians<br>**4.1.2.4** Physicians have to type their referrals themselves	**2.2.2.5** Saving time looking for patient records<br>**2.2.2.7** Physicians preferred EPR to paper records
Davidson 2007 [92]	**1.2.2** Reluctance to replace a recently acquired (or still functional) practice management system in order to integrate with an EHR<br>**1.4** Interfacing EHR software with various systems might create problems<br>**1.9** Physicians' perceptions of higher investment costs<br>**2.2.2.2** Uncertainty about selecting a vendor that might go out of business<br>**4.1.1.2** The cost and complexity of implementing an EHR in a multiple locations practice will be greater than in a single location practice<br>**4.1.2.4** The burden of revising work-flow processes might fall primarily on the physician, adding uncompensated hours<br>**4.1.4.3** Computer and network maintenance<br>**4.1.5.10** Deciding which EHR to adopt – or whether to it adopt at all – is a complex task for physicians<br>**4.2.1** Physicians' expectations for reduced costs in the future, due to subsidies from other health care players in the local market	**4.1.3.2** Leadership and advice

DesRoches 2008 [40]	**1.1** Concern that a system would become obsolete.<br>**1.2.3** Physicians expressed reservations about the ease of use of their systems<br>**1.3** Physicians expressed reservations about the reliability of their systems<br>**1.7.3** Incompatibility of the system with physicians' needs<br>**1.9:** Important capital costs and uncertainty about return on investment<br>**4.1.1.2** EHR adoption is more difficult for smaller practices	**1.5.2** Protecting physicians from personal liability for record tampering by external parties<br>**4.2.1** Financial incentives for EHR purchase
Gadd 2001 [82, 83, 86]	**1.5.1** Concerns that the EMR had a negative impact on patient privacy<br>**1.5.1** Personal and professional privacy concerns<br>**3.1.2** Using an EMR create a physical barrier causing the patient to be more distant<br>**4.1.2.1** Impact of the EMR on the time required to enter orders and document encounters	**3.1.2** Physicians stated that their patients appreciate that they had ready access to progress notes from previous visits<br>**4.1.2.3.1** Positive impact of EMR on quality and coordination of care, and communication within the health care team
Gans 2005 [62]	**1.9** Lack of capital resources to invest in an EHR<br>**1.12** Concern about loss of productivity during transition to an EHR system<br>**4.1.1.2** Smaller practices have lower EHR adoption rates, lower available financial resources and lower administrative capacity<br>**4.1.4.3** Lack of support<br>**4.1.5.10** Lack of the ability to evaluate EHR systems and inability to find systems that meet the practices' needs	**4.1.5.10** Educational programs on how to select and implement an EHR system and certification for EHR vendors
Hier 2005 & 2005 [65, 76]	**1.1** System speed<br>**1.2.3** Lack of understanding of EHR features<br>**1.5.1** Security concerns<br>**4.1.3.3** Lack of computer skills<br>**4.1.4.2** Lack of computers	
Jensen 2007 [91, 93]	**1.8** The surgeons expressed negative attitudes because they felt neglected in the decision process<br>**2.1.2** Lack of computer skills<br>**4.1.2.1** EPR use is time-consuming<br>**4.1.2.3.1** EPR does not support collaborative work<br>**4.1.2.4** EPR introduced new tasks that were not considered physicians' field of responsibility	**1.2.1** EPR eases work<br>**1.2.3** EPR system facilitates medicine prescription<br>**2.2.2.7** Easier ways of working fosters positive attitudes<br>**4.1.2.1** EPR allowed time-saving continuous access to patient information<br>**4.1.3.1** Super-users and secretaries are ambassadors of the project and their enthusiasm is important

	**4.1.5.3** Surgeons referred to the system as a control mechanism	
Keddie 2005 [67]	**1.5.2** Concerns about the medico legal implications of paperless practice<br>**1.9:** Issues of finance<br>**4.1.2.1** Lack of time<br>**4.1.4.3** Lack of technical support<br>**4.1.5.1** Lack of training	
Lium 2008 [46]	**1.1** Inability to high-light documents that carried important information and unplanned system downtime<br>**1.2.1** Initial reluctance regarding use of the system<br>**1.2.3** Lack of understanding of EHR features	**1.2.1** Physicians did not miss the time when they had only paper-based medical records and a paper-based workflow<br>**2.2.2.7** Understanding that the EMR had the potential of becoming a useful tool, and communication of this point of view throughout the organization<br>**4.1.2.4** Changes in admittance and discharge routines took advantage of the possibilities offered by the medication module and improved the ease of writing prescriptions and sick leave notes<br>**4.1.3.2** Clear lines of communication and decision-making; an excellent coordinator that kept staff informed and involved in the process<br>**4.1.4.3** IT departments demonstrated positive attitudes, willingness to go to great lengths and responded rapidly to requests from users<br>**4.1.5.3** Management required the physicians to use the system and the physicians obeyed. Physicians were well informed before the systems were implemented and their objections were listened to.<br>**4.1.5.5** Input from staff at all levels was accepted and appreciated<br>**4.2.2** The hospital began its approach by learning from the mistakes of other organizations
Ludwick 2009 [35]	**1.1** Need for more efficient data entry<br>**2.2.2.2** Technical support person did not know how a clinical practice functioned<br>**3.1.2** Exam room layouts [with a EHR on computer] compromised the quantity and quality of patient-physician information transfer<br>**4.1.1.4** The fee-for-service payment of primary care physicians contributed to limiting their time available for procuring and	

	implementing EMR<br>**4.1.4.3**. Insufficient and ill-timed training<br>**4.1.5.10** Physicians found the procurement process disorientating as they had no previous related experiences	
Menachemi 2006 [59]	**1.1** Inability of access to records if computer crashes<br>**1.7.3** Available products do not meet needs<br>**1.7.5** Lack of uniform data standards within the industry<br>**1.9** High costs of hardware/software and ongoing maintenance, resulting in an inadequate return on investment<br>**1.12** Temporary loss of productivity and/or revenue<br>**4.1.2.1** Lack of time to acquire/implement EHR and enter data	
Russell 2004 [71]	**1.5.1** Concerns about patient privacy<br>**3.1.2** Negative impact on doctor-patient interaction	
Sequist 2007 [55]	**1.1** Technical limitations of computers, such as slow response time<br>**1.12** Clinical productivity loss<br>**4.1.5.1** Need for additional training on appropriate methods of simultaneously interacting with the patient and the computer	
Simon 2007 [57]	**1.1** Technical limitations of systems<br>**1.5.1** Privacy or security concerns<br>**1.7.5** Lack of uniform standards<br>**1.9** Start-up and ongoing financial costs<br>**1.12** Loss of productivity<br>**4.1.1.2** Small practices are less likely to adopt EHR<br>**4.1.2.1** Lack of time to acquire knowledge<br>**4.1.3.3** Lack of computer skills<br>**4.1.4.3** Lack of technical support	**4.1.5.7** Organization influenced the decision of whether to adopt a new EHR system<br>**4.1.5.8** Availability of incentives for adoption of health information technology
Simon 2008 [45]	**1.7.3** Inability to find an EMR suited to needs<br>**1.9** Lack of adequate funding<br>**4.1.1.2** Solo practices had no plans to adopt EMRs but practices with 2–3 doctors and specialty practices were more likely to adopt<br>**4.1.2.2** Interference with workflow<br>**4.1.4.3** Lack of technical knowledge or support	
**Health professionals**		
Auber 2001 [85]	**1.2.5** Technology does not seem to improve the image of the health care professional	**1.2.1** Mandatory use of the Health Card by clients and the relative advantage for the professional

		**1.2.2** Compatability of the system with the work habits of the health care professionals<br>**1.2.3** Health Card system ranked high on ease of use<br>**1.7.1** High information quality<br>**1.7.2** Information was clear and easy to read<br>**1.12** The system improved communications and efficiency<br>**2.2.2.7** Marked positive attitude toward the Health Card, suggesting that respondents would continue to use it<br>**4.1.4.3** Professionals received diligent and efficient support<br>**4.1.5.3** Participants were not pressured to use their Health Card and used it voluntarily
Boulus 2007 [50]	**4.1.2.4** Introduction of a new function was rejected by staff, who perceived additional work tasks<br>**4.1.3.1** Users claimed their workload was so high that they could not adopt additional tasks<br>**4.1.5.3** Health care personnel did not always accept decisions regarding changes in work practice	**4.1.2.4** New functions were evaluated in relation to existing functions and the overall context of changes and workloads<br>**4.1.5.3** Evaluation-based decision-making<br>**4.1.5.4** Continuous discussions and negotiations among committee members<br>**4.1.5. 6** Establishment of the EPR committee and their meetings was a major driving force
Chronaki 2007 [49]	**1.5.1** Concern about security and confidentiality issues related to storage of personal data in the EHR<br>**3.1.2** Hesitation towards the use of a technology that could have a negative impact on the patient-physician interaction<br>**4.1.2.1** Heavy workload.<br>**4.1.4.3** Limited secretarial support and shortage of medical personnel	
Crosson 2005 [64]	**1.2.1** System of built-in reminders was disabled because the front desk supervisors assumed that “clinicians know what to do.”<br>**3.2.1** Resistance of the front desk supervisor<br>**4.1.2.3.1** Lack of practice-wide discussion regarding how the practice would use the EMR led to unforeseen consequences<br>**4.1.5.3** Hierarchal decision making: partners brought into the planning discussions only after lead physician had set long-term goals	**1.12** Partner in the practice saw the EMR as a tool to increase efficiency in the clinical encounter
Darbyshire 2004 [80]	**1.1** Recovered information was unusable<br>**1.2.3** Data entry and ease of use negatively perceived<br>**1.6** Inability to pull data out<br>**1.7.3** Practitioners believed their data entry efforts created only a	**1.2.1** Despite initial misgivings, after positive experiences the nurse perceived the usefulness of the system<br>**2.2.2.7** Evidence supported their needs<br>**4.1.2.1** Reduction of nurses’ time-consuming administrative

	partial view of their practice, both in quality and quantity<br>**2.2.2.6** No helpful influence in identifying or improving clinical outcomes<br>**4.1.2.1** System was difficult and time-consuming to use<br>**4.1.2.3.2** Clinical information was also viewed as belonging to the more powerful groups within the organization and thus not part of nurses' legitimate business<br>**4.1.2.4** Clinicians described entering "management's information" which they viewed as having little to do with clinical practice	tasks
Goddard 2001 [90]	**1.5.1** Confidentiality<br>**1.7.2:** Lack of basic data regarding clinical care<br>**2.2.2.7** Resistance to change<br>**4.1.5.3** Lack of organizational investment into technology and support services	
Greenhalgh 2008 [37]	**1.2.2** SCR use was poorly aligned with healthcare assistants' existing role, so accessing patients' SCRs proved difficult operationally<br>**1.2.3** Users perceived the SCR to be an immature technology<br>**1.4:** Poor interfacing with other information technology systems<br>**1.5.2** Concerns about the legality of the implied consent model<br>**1.11** Practitioners felt that their role was to "protect" their patients' data rather than to "pass it to the government"<br>**2.1.2** Limited success with implementation of the SCR was attributed to failure to appoint staff with key experience and qualities<br>**2.2.2.2** Poor linkage with the product's designers<br>**3.2.3** Organization was strongly opposed to the implied consent model and provided well argued resistance to it<br>**4.1.2.1** Workload was the main concern of general practices in early adopter sites<br>**4.1.2.4** Instructions were not adapted to change usual tasks<br>**4.1.4.3** Lack of staff to accomplish the required work<br>**4.1.5.1** Training was ill-timed or difficult to apply in practice<br>**4.1.5.2** Little in-house expertise in information technology existed<br>**4.1.5.3** No reflection on, or efforts to correct, underlying system level errors<br>**4.1.5.5** General practices and unscheduled car providers resented	**1.2.1** Manager linked the SCR to a wider organizational improvement strategy<br>**4.1.1.2** Implementation easier for larger practices as work can be divided among more staff<br>**4.1.2.3.2** People who worked in more than one organization cross fertilised ideas between them<br>**4.1.3.1** Opinion leaders travelled the country to explain what the SCR was, hear the concerns of their fellow, and make their audiences more receptive to the programme<br>**4.1.5.3:** Systematic and reflexive approach to data collection and analysis<br>**4.2.1** A financial incentive scheme to improved the quality of data<br>**4.2.2** Networking events to bring people from different early adopter primary care trusts together is needed

	being pushed to adopt primary care trusts<br>**4.1.5.9** Clinicians unwilling to assess patients without access to records<br>**4.2.3** Climate of uncertainty cited as a reason for not wishing to be involved in the programme	
Kossman 2006 [56]	**1.1** System speed and downtime interfered with nurses' ability to efficiently manage time<br>**1.12** EHR use caused frustration and a sense of decreased effectiveness in job performance and patient care<br>**3.1.2** Health professionals wanted to look at patient and not screen during assessments<br>**4.1.2.3.1** Limited communication among members as they did not read each others' notes in the EHR	**1.12** Quicker documentation and information retrieval processes
Laerum 2004 [88]	**1.1**Various software and hardware-related problems and system speed<br>**1.7.2** Inability to search images , which affects user satisfaction<br>**4.1.2.4** Tasks were more difficult to perform than before<br>**4.1.4.2** Lack of computers	**1.2.1** Defined tasks performed more easily than before<br>**1.12** Patient data more accessible
Likourezos 2004 [73]	**1.1** Confusing to follow the sequence of screens<br>**1.2.1:** Physicians report they are finish work more slowly than before<br>**1.5.1** A few clinicians stated that security of patient information is a concern they have with EMR systems.<br>**2.2.2.1** Will not make patient care less expensive<br>**2.2.2.5** Will not decrease patient waiting time<br>**2.2.2.6** Will not decrease the number of laboratory tests and the number of visits<br>**4.1.2.1** Amount of time required to perform a task	**1.2.1** A majority of nurses report that they are able to finish work faster and better monitor patient progress<br>**1.2.3** Physicians and nurses find it easy to enter data, access data, and read text on the screen<br>**2.2.2.6** Improved health care nursing<br>**2.2.2.7** Clinicians would like to computerize their medical records
Linder 2006 [54]	**1.1** Computers too slow, pop-up blocking software that interfered with the EHR and inability to access other functionalities while writing a note<br>**1.11** Concern about losing data<br>**2.1.2** Inability to type quickly enough<br>**3.1.2** Loss of eye contact with patient<br>**4.1.4.2** Lack of fast, available printers and lack of computers in some exam rooms	
Lium 2006 [60]	**1.2.1** Important tasks had become more cumbersome after the	**1.2.1** Tasks became more effective

	withdrawal of the paper medical record<br>**1.3** Delays or hindrances due to computer errors or the slow working of the system<br>**3.1.2** Nurses are primarily concerned with direct patient care and so they consider the EMR system to be interrupting rather than supporting their primary task	**2.2.2.7** Positive view of the effects of the EMR system for most tasks
Moody 2004 [70]	**1.1** Problems with the software and system and interruptions while documenting patient care<br>**4.1.4.2** Not enough space in patients' rooms to use the EHR	**2.2.2.6** Positive view of the impact of EHRs on patient care<br>**4.1.4.3** Help was always available
Ochieng 2006 [44]	**1.2.1** Computers are more beneficial for administrative than clinical functions<br>**1.9** Cost of computerisation is too high<br>**4.1.5.1** Training staff is too much effort	**2.2.2.7** EMR can significantly improve the quality of patient care
Ovretveit 2007 [51, 52]	**1.9** Disagreements about payment for the system<br>**2.2.4** Past experience was not positive<br>**4.1.4.3** Not allowing extra personnel time<br>**4.1.5.6** Difficulties involving doctors in the preparation work	**1.1** Potential for system development<br>**1.2.3** User friendly intuitive system needed little training<br>**1.2.4** Tried and tested system<br>**1.4** Order entry not difficult to integrate<br>**4.1.3.1** Competent IT project leader and team<br>**4.1.5.3** Prioritization and driving by management team<br>**4.1.5.5** Need for consultation before implementation<br>**4.1.5.6** Consensus about need for the system and which system was best
Rahimi 2008 [36]	**1.1** Logging on and calling up a specific file were time-consuming<br>**1.2.3** Functions were unintuitive and not user-friendly<br>**2.2.2.7** Unwillingness to adapt clinical routines to the new system<br>**4.1.2.1** Learning to use new terms and concepts took time<br>**4.1.5.1** Failure to give adequate training adjusted to the needs of nurses and other non-clinicians<br>**4.1.5.3** Policy-makers decided to implement the system in too short a time period<br>**4.1.5.6** More user participation in the design and implementation phase of the system would have provided a better fit into workflows and work practices	**4.1.4.3** Lack on ongoing support
Randeree 2007[48]	**1.1** Technology obsolescence and upgrades made old equipment incompatible<br>**1.5.1** Security issues<br>**1.5.2** The control of the patient data is also critical to adoption	**4.1.5.10** Standards for EMRs through certification make choosing a vendor easier

	**1.7.3** Inability to search imaged lab reports<br>**1.9** Maintenance agreements proved to be expensive<br>**1.12** Productivity decline and revenue loss during transition phase<br>**2.2.2.2** Lack of trust in vendor and deterioration in contract quality<br>**2.3.1** The older physicians were reluctant to transition to the EMR<br>**4.1.1.2** For small to medium sized practices without large IT budgets, costs remain the biggest barrier to adoption<br>**4.1.2.1** Time consuming to enter new and old patients into the system<br>**4.1.2.3.1** Using paper records caused some political infighting<br>**4.1.3.3** Staff were either unwilling or lacked the skill set to work with the new IT environment<br>**4.1.4.3** Poor vendor service and lack of support<br>**4.1.5.1** Staffing is a big concern since training takes time	
**Managers**		
Ferris 2009 [34]	**1.1** The system displays and alerts were sometimes incorrect<br>**1.2.2** The formatting of patient letters generated through ERM was not suited to the clientele<br>**1.2.3** Confusion regarding how to view all results for a particular individual<br>**1.4** Flaws with external connectivity<br>**4.1.5.1** Deficiencies in ERM training and follow-up assistance	**1.2.2** The ability to generate well-formatted letters rapidly from the application is an important gain in efficiency
Houser 2008 [42]	**1.5.1:** Privacy issues<br>**1.9:** Lack of adequate funding and resources<br>**2.1.2** Lack of knowledge of EHRs<br>**4.1.1.1** Hospital location<br>**4.1.1.3** No differences in hospital bed size or teaching status in relationship to EHR implementation status<br>**4.1.4.3** Lack of support from medical staff<br>**4.1.5.1** Lack of employee training	**1.9** Reduced costs<br>**2.2.3.4** Reduced medical errors and treatment time<br>**2.2.3.6** Improvements in workflow<br>**2.2.3.7** Improvements in clinical processes or workflow efficiency
Lorence 2005 [63]	**1.7.4** Only half of respondents believed the accuracy of patient data is better with computerized records<br>**1.7.5** Need for standards to facilitate the exchange of health-care data<br>**2.1.1** Unfamiliarity with data-driven medical decision-making<br>**4.1.4.3** Shortages of trained health information professionals	

	**4.1.5.1** The involvement of health-care professionals who are familiar with paper-based information management	
Mannan 2006 [58]	**1.5.1** Concerns that unauthorised personnel would gain access to patient records<br>**1.9** Costs associated with the National Programme for IT<br>**4.1.5.10** Difficulty finding a system that has proven its effectiveness	
Martin 2007 [53]	**1.2.2** Compatibility with work habits<br>**1.5.1** Since it is proposed that terminals will be placed in public areas, not logging out represented a security risk<br>**4.2.2** The new system had to integrate with NHS requirements and be used to present the hospital work statistics	**2.2.3.6** No clear understanding of the best outcome
Miller 2004 [78]	**1.2.3** EMRs are challenging to use due to the multiplicity of screens, options, and navigational aids<br>**1.4** Lack of adequate electronic data exchange between the EMR and other clinical data systems<br>**1.9** The high up-front financial cost<br>**4.1.1.2** Physicians in solo/small-group practice<br>**4.1.2.1** The extra time necessary to learn how to use the EMR effectively for daily tasks<br>**4.1.2.4** Additional tasks<br>**4.1.4.3** Lack of vendor or internal IT support<br>**4.1.5.8** Financial incentives for quality	**1.1** EMR usability
Scott 2005 [61]	**1.1** Software problems<br>**1.9** Higher than anticipated implementation costs<br>**1.12** Reductions in clinician productivity<br>**4.1.2.4** Extra work due to processing laboratory result reports, entering orders, and navigating through the system<br>**4.1.2.5** Hawaiians are perceived as averse to conflict and likely to interpret negative feedback as personal criticism<br>**4.1.4.3** Lack of clinical capacity to absorb changes during implementation.<br>**4.1.5.3** Consensus seeking encouraged passive resistance<br>**4.1.5.5** Conflicting priorities between the organisation as a whole and individual clinicians<br>**4.1.5.6** Adequate clinician participation in decision-making<br>**4.1.5.10** Disagreement with the chosen system	**4.1.2.4** Greater accountability

Thakkar 2006 [47]	**1.4** Lack of interoperability among different electronic systems and the true EHR system.<br>**1.7.5** Lack of national information standards and code sets<br>**1.9** Software costs, hardware costs, return on investment and personnel cost<br>**4.1.1.2** The automation of work flow offered through an EHR system does not provide as much benefit when the size of the hospital increases<br>**4.1.5.2** Organizational culture<br>**4.1.5.6 Lack of** participation from nursing staff<br>**4.1.5.10** Inability to find software that meets needs	**1.4** Interoperability with other departments within the facility<br>**1.5.1** Patient privacy and confidentiality<br>**1.9** Reduced costs of care<br>**1.12** Medical staff's work efficiency and time management<br>**2.2.x.7** Improvement of quality of care<br>**3.1.2** Patient-doctor relationship<br>**4.1.2.1** Clinical workflow
Urowitz 2008 [39]	**1.9** Hospital financial resources<br>**2.1.2** Computer literacy	
Yasunaga 2008 [43]	**1.9** Few institutions perceived EMR as cost-effective<br>**4.1.1.1** Institutions with a fewer number of beds had lower adoption rates<br>**4.1.1.3** Private hospitals had lower rates of adoption<br>**4.1.2.1** Increased workload of doctors	**1.12** Increased time efficiency and information sharing
**Patients**		
Bomba 2001 [84]	**1.3** Computers perceived as less secure and reliable than traditional paper based information systems<br>**1.5.1** Concerns about privacy and security of health information<br>**2.2.1.1** Belief that the risks overweigh the benefits of the system<br>**4.2.3** Lack of trust in government organization	**1.7.4** Easy access to accurate and useful data.<br>**2.2.1.1** Belief that computers have the potential to improve the quality of health care received at a medical practice<br>**2.2.4** Belief that computer based patient records is an essential technology for health care in the future.<br>**3.1.2** Use of computers did not interfere with the consultation
Dagnone 2006 [89]	**1.3** Potential for the technology to "crash"	**1.2.1** Reduce the problems of deciphering poor handwriting<br>**1.3** Decrease the likelihood of data loss or data entry errors<br>**1.7.4** Improve of the accuracy of the medical record<br>**1.12** Increase the efficiency of patient assessments<br>**2.2.1.1** The use of portable computers at the bedside was satisfactory or superior to the conventional paper chart assessment<br>**2.2.1.7** Patient interest in the implementation of this technology in the hospital setting<br>**3.1.2** The use of a portable electronic device at the bedside did not affect the clinician-patient relationship

		**4.1.2.1** Reduction in the workload of clinicians<br>**4.1.5.6** Higher degree of involvement in the assessment process because of personal curiosity about the technology
Greenhalgh 2008 [41]	**1.2.1** Limited capacity to understand<br>**1.5.1** Security concerns<br>**1.5.2** Fear that the government would be tempted to make money from a range of secondary uses<br>**2.1.1** Unawareness of EHR<br>**2.2.1.1** Unfavourable risk-benefit equation<br>**2.2.1.4** Misunderstanding of SCR content<br>**2.2.1.6** Belief that difficulties will be frequent with shared electronic records<br>**3.1.2** The SCR and Health Space were seen as potentially able to legitimise the patient's account of reality in situations where clinician trust was low	**1.2.1** Having information in one place is facilitating<br>**2.2.1.6** Allowing more time for important tasks<br>**2.2.4** Positive opinions about SCR
Hassol 2004 [77]	**1.7.4:** Inaccurate medication list	**1.2.3:** Ease of use<br>**1.5.1:** Lack of concern about security and confidentiality<br>**2.1.2:** Ability with computers and the internet
Honeyman 2005 [68]	**2.2.1.7:** Lack of interest and motivation	**1.5.1:** Confidence in the security of electronic record<br>**1.7.4:** Accuracy of electronic records<br>**2.1.2:** Ease of access to electronic records<br>**2.2.1.7:** Positive attitude toward the ability to access health records
Keselman 2007 [38]	**1.4:** Ease of access<br>**1.5.1:** Privacy, security, and confidentiality issues<br>**1.7.3:** Difficulty with medical terminology	**2.2.1.3:** System encouraged participants to take an active role in managing their own health.<br>**2.2.1.7:** Access to detailed health information and ability to check the accuracy of the record
Morin 2005 [66]	**1.3:** Concern over possible errors	**1.2.1:** Perceptions of a more systematic follow-up<br>**1.4:** Rapid exchange of information between professionals and healthcare facilities<br>**1.5.1:** Confidence in the security of the records<br>**1.7.2:** Rapid and complete access to information<br>**1.7.4:** Reduced risk of error<br>**1.12:** Facilitation of work for the healthcare professionals<br>**3.1.1:** Belief that computerisation is unavoidable and would not have a negative impact on the quality of data
Pyper 2004 [72, 79,	**1.5.1:** Concerns about confidentiality and security	**1.2.1:** Usefulness of record summaries and consultation details

81]	**1.9:** The cost of introducing electronic records and keeping technology up to date<br>**3.1.2:** Improvements in doctor–patient relationship, improvements of consultations, and encouragement of patients to be better informed about their own health and health care	**1.2.3:** Ease of navigation within the system<br>**1.5.1:** Consent for access the electronic record<br>**1.7.3:** Ease of understanding of EHR content<br>**1.7.4:** Improved accuracy and avoiding the need to read doctors' handwriting.<br>**2.1.2:** Computer skills<br>**2.2.1.1:** Positive attitude toward EHRs and their advantages<br>**2.2.1.3:** Increased patient involvement in decision-making about their own healthcare
Zurita 2004 [75]	**1.1:** Technical breakdown<br>**1.4:** Accessibility of information in the EHR to families of patients, doctors and hospitals<br>**1.7.4:** Errors in EHR	**1.5.1:** Respect of patient privacy<br>**2.2.1.7:** Positive attitude toward the use of EHR<br>**3.1.1:** Patients wanted the information be available for themselves
